# Application of Dimethicone to Prevent Culture Media from Drying in Microbiological Diagnostics

**DOI:** 10.17691/stm2023.15.1.02

**Published:** 2023-01-28

**Authors:** T.A. Savinova, Y.A. Bocharova, N.A. Mayansky, I.V. Chebotar

**Affiliations:** Leading Researcher, Laboratory of Molecular Microbiology; Pirogov Russian National Research Medical University, 1 Ostrovityanova St., Moscow, 117997, Russia; Senior Researcher, Laboratory of Molecular Microbiology; Pirogov Russian National Research Medical University, 1 Ostrovityanova St., Moscow, 117997, Russia; Professor of the Russian Academy of Sciences, Head of the Center for Laboratory Diagnostics, Russian Children Clinical Hospital; Pirogov Russian National Research Medical University, 1 Ostrovityanova St., Moscow, 117997, Russia; Head of the Laboratory of Molecular Microbiology; Pirogov Russian National Research Medical University, 1 Ostrovityanova St., Moscow, 117997, Russia

**Keywords:** bacteria growth and generation, bacterial mobility, culture media, microorganism cultivation, dimethicone

## Abstract

The search for novel modifications of culture media aimed at culture prolongation is a prerequisite for microbiological diagnostic progress.

**The aim of the study** was to assess the possibilities of applying dimethicone (polymethylsiloxane) as a barrier between the agar surface and atmosphere to prevent drying of solid and semisolid culture medium providing the retention of its useful properties.

**Materials and Methods:**

We studied the dynamics of water (volume) loss of culture media used in microbiology, and the effect of dimethicone on the process. Dimethicone was arranged in layers on culture medium surface. The effect of dimethicone on growth and generation of fast-growing (*Staphylococcus aureus*, *Escherichia coli*, *Salmonella enterica* Serovar Typhimurium, *Burkholderia cenocepacia*) and slow-growing (*Mycobacterium avium*) bacteria was studied, as well as on bacterial mobility (*Pseudomonas aeruginosa* and *Escherichia coli*) in semisolid agars.

**Results:**

The dynamics of water loss in culture media showed the weight loss in all media without dimethicone (control) in 24 h to be statistically significant (p<0.05); 7–8 days later, they lost 50% of weight, and 14 days later they lost approximately 70%. The weight of media under dimethicone underwent no significant changes during the observation period. Growth index of fast-growing bacteria (*S. aureus*, *E. coli*, *S.* Typhimurium, *B. cenocepacia*) on control culture media without applying any substance, and on culture media under dimethicone had no significant differences. Visible *M. avium* growth on chocolate agar in controls was recorded on day 19, under dimethicone — on days 18–19. The number of colonies on culture day 19 under dimethicone tenfold exceeded the control values. The mobility indices of *P. aeruginosa* and *E. coli* on semisolid agar under dimethicone 24 h later were significantly higher than under control conditions (p<0.05 in both cases).

**Conclusion:**

The study confirmed marked deterioration of culture media properties under prolonged cultivation. The suggested protection technology of culture media growth properties using dimethicone showed beneficial effects.

## Introduction

Accurate identification of an agent (or agents) is a key condition for successful diagnosis and therapy of infectious diseases. The quality of microbiological diagnosis is determined by the entire spectrum of microorganism species found when studying biological material. The complete detection of microbes depends not only on the correct selection of culture media but also culture conditions including temperature, atmospheric composition, and duration. These factors gain particular importance in case microorganism cultivation is prolonged, which is required for qualitative diagnosis of some infectious diseases.

Human pathogens can be conditionally distinguished into fast- and slow-growing. Initially, such division was suggested for non-tuberculous mycobacteria in the second half of the XX century [1]. Bacteria are considered to be referred as slow-growing if they show visible growth within the period of over 7 days. There are other interpretations of the term “slow growth” based on determining the cell reproduction rate in liquid medium. However, in clinical practice reproduction, rate measuring techniques in broth are not very frequently used, therefore, culture methods using conventional culture media are more relevant in medicine. Currently, the term “slow-growing” is used both for mycobacteria and also bacteria from other taxons. For instance, *Mycoplasma spp*. bacteria show visible growth on agar media within the period up to 14 days [2]. Moreover, slow growth can be typical for strains within a single species belonging to typical fast-growing bacteria. Examples of such strains can be different bacterial forms with reduced or arrested metabolism, they are resulting from adaptation to antimicrobials [3].

Proper detection of a complete spectrum of bacteria on culture media can require a long-term culture. Moreover, prolonged incubation is used in a number of scientific techniques, e.g. when studying flagellar bacteria mobility in semisolid agars.

In practice, long-term culture on solid culture media is complicated by technical challenges. The major problem is solid culture medium drying, it resulting from syneresis (or water loss in gel) leading to the reduced volume and growth deterioration of medium. Drying can cause distortion of microbiological analysis findings [4], therefore, long-term culture generally require media with water-retaining components (glycerol, high concentrated proteins, and others), or culture under high humidity [5]. We consider the most promising approaches to prevent solid culture media from drying those based on barriers blocking evaporation between agar surface and atmosphere.

**The aim of the study** was to assess the possibilities of applying dimethicone (polymethylsiloxane) as a barrier between agar surface and atmosphere to prevent drying of solid and semisolid culture medium providing the retention of its useful properties in order to improve the quality of microbiological diagnostics.

## Materials and Methods

### Study design

The investigation included four stages. At the first stage, the dynamics of water weight (mass) by culture media frequently used in microbiological practice was studied, as well as the effect of dimethicone water-proofing fluid on the process; dimethicone was arranged in layers on culture medium surface. The second stage was the search for proofs that dimethicone has no inhibitory effect on growth and generation of essential fast-growing bacteria (*Staphylococcus aureus*, *Escherichia coli*, *Salmonella enterica* Serovar Typhimurium, *Burkholderia cenocepacia*) on conventional culture media including selective and chromogenic agars. At the third stage, we studied the growth and reproduction capabilities of slow-growing bacteria (using *Mycobacterium avium* as a model) under prolonged culture under dimethicone. During the fourth stage, we assessed the effect of dimethicone on bacterial mobility (*Pseudomonas aeruginosa* and *Escherichia coli*) in semisolid agars.

### Assessment of water loss dynamics in culture media

Water loss was assessed by determining the changes in water weight for the following media: Endo (Liofilchem S.r.l., Italy), UriSelect 4 Agar (Bio-Rad Laboratories Inc., USA), CHROMagar B. cepacia (CHROMagar, France), chocolate agar based on Levinthal medium (HiMedia Laboratories Pvt. Ltd., India). Dimethicone, 9.0 ml (XIAMETER™ PMX-200 Silicone Fluid 350 cSt; Dow, USA), was arranged in layers on culture media surface in a Petri dish, 90 mm in diameter. As controls, we used native culture media without applying any substance. Culture medium weight was first measured 6 h after preparation, subsequent measurements were made every 24 h for 14 days. Petri dishes with media were kept in temperature-regulated chamber at 35°C. The results were represented in percentage of medium weight, according to the first weighing.

### Bacterial strains

In the study, reference strains from American Type Culture Collection (ATCC) were used: *Escherichia coli* (ATCC 25922), *Pseudomonas aeruginosa* (ATCC 27853), *Salmonella enterica* Serovar Typhimurium (ATCC 14028), *Staphylococcus aureus* (ATCC 29213). Strain *Burkholderia cenocepacia* 182/2 had clinical origin, its genome was registered in the GenBank database (accession number JAKCPP000000000). Strain *Mycobacterium avium* 018 was a clinical isolate, its species belonging was proved using MALDI-TOF mass spectrometry (Microflex LT spectrometer, flexAnalysis 3.0 and MALDI Biotyper 3.0 Offline Classification software; Bruker Daltonik GmbH, Germany).

### Inoculation and cultivation

*S. aureus* and *E. coli* were cultured in mixed culture on medium UriSelect 4 Agar within 24 h. *E. coli* and *S*. Typhimurium were cultured in mixed culture on Endo medium within 24 h. *B. cenocepacia* 182/2 was cultured on CHROMagar B. cepacia medium within 48 h. *M. avium* 018 was cultured on chocolate agar till the colonies visible with the naked eye, but for no more than 20 days.

For inoculation, we used suspensions of bacteria standardized by optical density. Suspensions with 0.5 McFarland turbidity were dissolved by 10,000 times by normal saline. Inoculation on Petri dishes (diameter of 90 mm) was performed using an automatic device for culture EasySpiral® Pro (Interscince, France) in the exponential spiral mode and dosage of 50.0 μm. After inoculation, the dishes were kept for 15 min at room temperature. Then, 9.0 ml of dimethicone was applied by layers on the central part of culture medium surface, spreading evenly on the dish surface; the total dimethicone layer thickness was approximately 1.5 mm.

As controls, we used: 1) dishes with culture without any substance applied (hereinafter — control); 2) similar to dimethicone, mineral oil (Microgen Bioproducts Ltd., Great Britain) was applied in layers on culture medium. Inoculation was performed in natural atmosphere, at 35°C. Each experiment was repeated in triplets at least three times.

### Study of dimethicone effect on bacterial mobility

The effect of dimethicone on bacterial mobility was assessed by growth area changes of *E. coli* and *P. aeruginosa* on semisolid agar. Semisolid agar was based on Difco LB Broth, Lennox (Becton Dickinson and Company, USA) containing 0.26% of bacteriological agar (Liofilchem S.r.l., Italy). Suspended bacteria, 0.1 ml (*E. coli* or *P. aeruginosa*), their concentration being approximately 2**·**103 cells/ml, was injected in Petri dish center, 90 mm in diameter, with 25.0 ml of semisolid agar. Dimethicone, 9.0 ml, was applied in layers on the culture medium center; the samples without any substance applied were used as controls.

### Evaluation of findings

*S. aureus*, *E. coli*, and *S*. Typhimurium growth results were assessed after 24 h, *B. cenocepacia* 182/2 — after 48 h. *M. avium* growth was recorded every 24 h till it showed visible growth; when visible colonies appeared, their numbers were counted.

The mobility of *E. coli* and *P. aeruginosa* was assessed after 24 h by determining their growth area.

***The data were statistically analyzed*** using the SPSS 20.0 software (IBM SPSS Statistics, USA). Shapiro– Wilk W test was used to assess data distribution. In case of normal distribution, arithmetic mean and standard deviation (M±σ) were determined. In case the distribution differed from normal, the mean values were represented in the form of medians and values of 25th and 75th quartiles (Me [25%; 75%]), Mann–Whitney U test was used to determine statistical significance of differences. The differences were considered statistically significant if p≤0.05.

## Results

Table 1 shows the dynamics of water loss by culture media. A day later, the weight decrease for all media without dimethicone (control) was statistically significant (p<0.05). On days 7–8, the media in control experiments lost 50% of weight. In 14 days, the control media showed 70% weight reduction. The weight of media under dimethicone showed no significant decrease during the observation period, it changed at the end of the period only, the decrease amounting to 3% (p>0.05).

**Table 1:** Water loss dynamics by culture media

Medium type, experimental conditions		Culture medium weight (percentage of medium weight at the first weighing, M±σ) at different keeping time (days)														
		After 6 h	Day 1	Day 2	Day 3	Day 4	Day 5	Day 6	Day 7	Day 8	Day 9	Day 10	Day 11	Day 12	Day 13	Day 14
Endo	Control	100	94.7±0.8	85.8±1.2	77.6±2.3	70.8±3.7	64.4±3.6	59.4±3.3	53.9±4.3	50.4±0.7	45.9±1.7	40.8±1.4	36.0±1.7	33.5±1.6	32.1±0.5	31.4±0.3
	Dimethicone		100.0±0.1	100.0±0.1	100.0±0.1	100.0±0.2	100.0±0.1	100.0±0.1	100.0±0.1	100.0±0.1	100.0±0.3	100.0±0.3	100.0±0.4	100.0±0.3	98.1±1.3	98.4±1.1
UriSelect 4 Agar	Control	100	95.2±0.9	86.3±1.1	79.5±2.4	73.2±4.9	67.1±5.3	61.5±3.0	55.4±3.3	49.9±4.0	44.4±4.0	39.0±2.7	34.8±2.2	30.2±0.2	29.4±0.3	28.7±0.1
	Dimethicone		100.0±0.1	100.0±0.1	100.0±0.1	100.0±0.1	100.0±0.1	100.0±0.1	100.0±0.1	100.0±0.1	100.0±0.3	100.0±0.3	100.0±0.8	98.8±1.5	98.0±1.0	97.1±1.7
CHROMagar B. cepacia	Control	100	94.4±0.7	87.4±1.3	80.7±3.4	74.4±5.3	68.0±5.3	62.7±4.2	57.2±4.4	52.4±3.4	46.8±3.3	40.5±2.8	34.4±2.3	32.2±1.0	30.9±0.5	30.5±0.3
	Dimethicone		100.0±0.1	100.0±0.1	100.0±0.2	100.0±0.2	100.0±0.2	100.0±0.2	100.0±0.2	100.0±0.2	100.0±0.2	100.0±0.9	100.0±1.5	99.2±1.0	99.0±0.9	99.2±1.3
Chocolate agar	Control	100	94.8±0.3	90.4±1.2	86.5±5.4	80.5±7.4	74.6±6.9	68.2±6.4	62.2±5.6	56.0±5.4	48.1±4.2	42.2±4.3	36.1±3.1	34.1±1.3	29.9±0.8	29.2±0.4
	Dimethicone		100.0±0.1	100.0±0.1	100.0±0.1	100.0±0.1	100.0±0.1	100.0±0.1	100.0±0.1	100.0±0.1	100.0±0.2	100.0±0.4	100.0±0.4	98.1±1.5	98.1±1.4	97.4±1.2

Growth indices of fast-growing bacteria (*S. aureus*, *E. coli*, *S*. Typhimurium, *B. cenocepacia* 182/2) on culture media without any substance applied and on media under dimethicone had no significant differences (Table 2).

**Table 2:** Bacterial growth indices on solid culture media without dimethicone applied (control) and with dimethicone application

Culture types	Experimental conditions	Type	Number of colonies, Me [25%; 75%]	Statistical significance of differences with controls
Mixed culture *E. coli* (ATCC 25922) + *S. aureus* (ATCC 29213) on UriSelect 4 medium	Control	*E. coli*	87 [75; 115]	NA
		*S. aureus*	89 [73; 117]	NA
	Dimethicone	*E. coli*	92 [75; 110]	p>0.05
		*S. aureus*	97 [86; 118]	p>0.05
Mixed culture *E. coli* (ATCC 25922) + *S*. Typhimurium (ATCC 14028) on Endo medium	Control	*E. coli*	110 [87; 140]	NA
		*S.* Typhimurium	155 [117; 192]	NA
	Dimethicone	*E. coli*	101 [73; 137]	p>0.05
		*S.* Typhimurium	158 [110; 201]	p>0.05
Monoculture *B. cenocepacia* 182/2 on CHROMagar B. cepacian medium	Control	*B. cenocepacia*	501 [413; 596]	NA
	Dimethicone	*B. cenocepacia*	487 [390; 564]	p>0.05
Monoculture *M. avium* 018 on chocolate agar	Control	*M. avium*	21 [11; 29]*	NA
	Dimethicone	*M. avium*	280 [230; 311]*	p<0.05

Note: NA — not applicable; * findings on day 19.

The Figure represents standard growth images of fast-growing bacteria, both in controls and under dimethicone.

The experiments with the medium of mineral oil applied in layers used as control showed the oil to block bacterial growth completely: there were no signs of *S. aureus*, *E. coli*, and *S*. Typhimurium colonies on culture media within 24 h, *B. cenocepacia* 182/2 — within 48 h, *M. avium* 018 — within 20 days.

In 7 days, control dishes with *M. avium* without any substance applied showed the visible signs of chocolate agar drying. Visible *M. avium* growth in control was recorded on day 19. Visible growth was observed on agar under dimethicone on days 18–19. The number of colonies 19 days later on the medium under dimethicone increased tenfold compared to the controls (see Table 2).

The growth area of *P. aeruginosa* on semisolid agar in control in 24 h was 22.1±9.3 cm2, and under dimethicone Petri dish surface (63.6 cm2) was overgrown completely (Figure (c), (g)). The values had statistically significant differences (p<0.05). The growth area of *E. coli* on semisolid agar in control in 24 h was 1.1±0.5 cm2, while under dimethicone it was 3.9±0.9 cm2 (Figure (d), (h)), the values had statistically significant differences (p<0.05).

**Figure:**
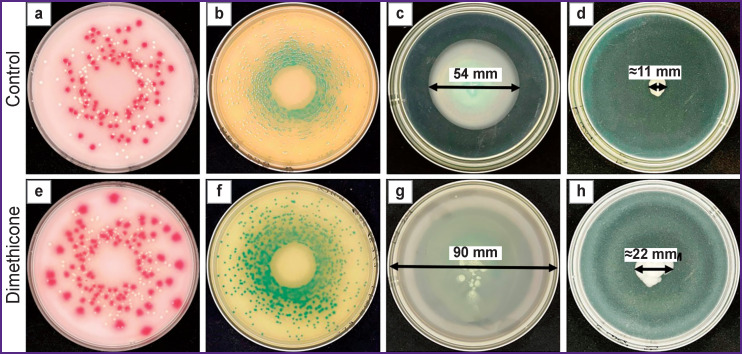
Standard images of bacterial growth on culture media under dimethicone layer and without its application: (a)–(d) growth without dimethicone depositing (control), (e)–(h) growth under dimethicone; (a), (e) 24-h culture *E. coli* (*pink colonies*) and *S. aureus* (*white colonies*) on medium UriSelect 4 Agar; (b), (f) 48-h culture *B. cenocepacia* on medium CHROMagar B. cepacia; (c), (g) and (d), (h) 24-h culture *P. aeruginosa* and *E. coli*, respectively, on semisolid agar; arrows show the diameter of bacterial growth zone

## Discussion

The obtained results demonstrate three useful characteristics of dimethicone for practical microbiology.

Firstly, dimethicone blocks culture media drying, it relates to its high hydrophobic property and relatively low density (969 kg/m3) [6]. Due to these characteristics, dimethicone does not dissolve in water-based culture media and it can stay above their surface for a long time. Dimethicone makes a hydrophobic barrier for water evaporation at the borderline between the media and atmosphere. Exhibiting low volatility, dimethicone can preserve its weight under thermostatic control at temperature up to 45°C for a long time.

Secondly, dimethicone does not inhibit growth and reproduction on solid culture media of microbes, which were assayed in the present study. Moreover, under prolonged culture of mycobacteria on media under dimethicone protection, there was the best microbial growth compared to standard media. The result can be due to several reasons including preserving stability of rheological properties and the proportions of soluble and insoluble components formulating a culture medium; dimethicone capability to provide gas exchange between microbial cells and the environment; non-toxicity of polymethylsiloxanes in regard to living cells. The ability to dissolve oxygen, carbon dioxide, hydrogen and other gases is the key characteristic of dimethicone [7, 8]. Oxygen solubility in dimethicone by its weight hundredfold exceeds oxygen solubility in water. Due to high solubility of gases, dimethicone provides quick removal of gaseous wastes, which enable to inhibit generation of bacteria, and for aerobic bacteria — complete oxygen delivery. Non-toxicity of polymethylsiloxanes is proved by the fact they are used as components of cosmetic products and pharmaceuticals [9, 10]. Our findings of bacterial growth under dimethicone generally correlate with the data by Lam et al. [11], who previously used dimethicone in microfluid chips when culturing bacteria and mammal cells.

The third useful characteristic of dimethicone concerns its capability to be used to study the mobility of flagellar bacteria in semisolid media. Outrunning growth of *P. aeruginosa* and *E. coli* under dimethicone is likely to result from preserving rheological properties of culture medium gel. Growth retardation of bacteria on control media was related to drying and thickening of agar overlay media.

When discussing the findings, it is necessary to pay attention to high drying rate again: by day 8, media lost at least 50% of weight, and in 2 weeks — about 70%. This fact means that the proportions of medium components tragically altered leading to the deterioration of its growth properties; in practice, this implies mistakes in microbiological diagnostics. It substantiates the significance of developing novel techniques aimed at preserving culture media characteristics in long-term culture of microbes. The application of dimethicone is a promising method of microbiological diagnostics.

## Conclusion

The search for novel modifications of culture media aimed at culture prolongation is a prerequisite for microbiological diagnostic progress. The suggested technology to preserve growth properties of culture media using dimethicone demonstrated good results. The investigations carried out will be used for further study of properties of other substances belonging to polyorganosiloxanes, which can be useful to culture various microorganisms.
